# Effect of Low-Temperature Plasma Activated Water with Different Treatment Times on Myofibrillar Proteins of Thawed Pork

**DOI:** 10.3390/foods14060970

**Published:** 2025-03-12

**Authors:** Manting Du, Fangge Hao, Shunyang Sun, Ke Li, Qisen Xiang, Junguang Li, Lichuang Cao, Yanhong Bai

**Affiliations:** 1 College of Food and Bioengineering, Zhengzhou University of Light Industry, Zhengzhou 450001, China; mantingdu@163.com (M.D.);; 2 Key Laboratory of Cold Chain Food Processing and Safety Control, Ministry of Education, Zhengzhou University of Light Industry, Zhengzhou 450001, China

**Keywords:** plasma-activated water, thawed pork, myofibrillar proteins, gelation properties

## Abstract

In this study, myofibrillar proteins (MPs) of thawed pork were treated with plasma-activated water (PAW) generated at different times (0, 5, 10, 15, 20, and 25 s) to investigate whether the function of MPs is improved through PAW and the corresponding regulatory mechanism. The results found that PAW treatments increased the surface hydrophobicity and altered the secondary and tertiary structure of MPs. The α-helix content of MPs treated by PAW reduced from 37.3% to 31.25%. In the PAW25s group, the oxidation of MPs was significantly raised, reflected by the higher carbonyl content and lower total sulfhydryl content compared with other groups (*p* < 0.05). Furthermore, PAW treatments increased the whiteness and improved the strength, immobilized water contents, resilience, chewiness, and adhesiveness of MP gels. The observation of intermolecular forces and microstructure of MP gels presented an increase in ionic bonding, disulfide bonding, and hydrophobic interactions but a decrease in hydrogen bonding in MP gels with PAW treatments, leading to more homogeneous and denser gel structures compared with the control group. In conclusion, PAW, with a short generation time, significantly fixed and enhanced the function of MPs extracted from thawed pork and, to some extent, improved the processing quality of the MPs of thawed pork.

## 1. Introduction

Freezing serves as an essential way to extend the shelf life of meat products. It delays the deterioration of meat quality, reduces biochemical reactions, and inhibits microbial growth in meat [1]. However, during the freezing process, the majority of water in the pork turns into ice crystals, which severely damages the tissue structure of the pork, leading to the loss of thawing juices and the further deterioration of the quality of the pork [2]. After thawing, the internal environment of meat changes due to the damaged muscle cells and muscle fibers, causing changes in the pH and ionic strength of meat and finally damaging myofibrillar proteins (MPs). Consequently, the development of new technologies to improve the structure and functions of MPs of thawed meat will have a profound impact on the better utilization of thawed meat.

Low-temperature plasma-activated water (LT-PAW) is usually generated using a plasma jet gun charging directly under ionizing distilled water with high pressure or treating distilled water indirectly using dielectric blocking discharge (DBD), sliding arc, and other media [3]. LT-PAW contains a lot of active substances, including NO^2−^, NO^3−^, H_2_O_2_, and reactive oxygen and nitrogen species (RONS) [4,5]. In recent years, LT-PAW has gradually been applied to the food industry, particularly in food preservation and sterilization. Li et al. [6] investigated the effect of PAW on the aggregation degree and gelation properties of myofibrillar proteins of Aristichthys nobilis and found that PAW significantly enhanced the hydrophobic interactions of MPs but weakened ionic bonding, aggregation, and gelation of MPs. The structure of MPs is changed using the PAW treatment, and finally, the three-dimensional network structure and the gelation density of MP gels are improved. Qian et al. [7] prepared MP gels with PAW and discovered that the gels treated by PAW had intrinsic antimicrobial activity.

Currently, research on LT-PAW mainly concentrates on the sterilization and preservation of noodles, vegetables, and meat products [8]. The potential ability of LT-PAW to change the functions of MPs remains in development. In addition, the problem of the decrease in the MP properties of thawed meat still needs more solutions. Thus, this study aimed to verify the effect of LT-PAW on the MPs of thawed pork and illustrate the relative mechanism by evaluating the changes in structure and gelation properties of MPs after PAW treatments, expecting to provide theoretical guidance for the application of LT-PAW in improving the processing quality of thawed pork.

## 2. Materials and Methods

### 2.1. Materials

The *longissimus lumborum* muscles of 3 pigs were sourced from the local farmer’s market in Zhengzhou City and transported to laboratory in a temperature range of 0 to 4 °C. After transportation, the epimysia connective tissue and visible fat of muscles were removed and then the meat was stored at −20 °C for 72 h. All analytical grade chemicals were purchased from Zhengyong (Zhengyong Chemical Reagent Co., Ltd., Zhengzhou, China).

### 2.2. Preparation of PAW

Plasma generator was used to prepare PAW as described by Qian et al. [7]. PBS (0.6 mol/L NaCl, pH 7.0) buffer was pre-cooled to 4 °C in advance and divided into 6 portions of 300 mL each. PAW was obtained by the generation of plasma discharge over the surface of pre-cooled PBS for 0 s, 5 s, 10 s, 15 s, 20 s, and 25 s, respectively. The distance between the end of the plasma generator and the pre-cooled PBS surface was 2 cm.

### 2.3. Extraction of MPs and PAW Treatment

MPs were extracted as described by Zhu et al. [9] with slight modifications. Firstly, the thawed pork was minced using a meat grinder and added with 4 times volume (m:v) of extraction solution 1 (10 mmol/L Na_2_HPO_4_/NaH_2_PO_4_, 0.1 mol/L NaCl, 2 mmol/L MgCl_2_, 0.1 mol/L EGTA, pH 7.0, 4 °C). Then, the mixture was homogenized under the condition of 10,000 r/min for 2 min. The homogenate was centrifuged at 4 °C, 3000× *g* for 15 min, and the supernatant was discarded, and this was repeated three times. After that, the obtained sediment was mixed with 4 times the volume (m:v) of 0.1 mol/L NaCl wash solution, homogenized and centrifuged three times using previous conditions to obtain purified MPs.

MPs concentration was measured using the Biuret method and adjusted with PBS buffer (50 mmol/L Na_2_HPO_4_/NaH_2_PO_4_, 0.6 mol/L NaCl, pH 7.0, 4 °C). Subsequently, the prepared 0 s, 5 s, 10 s, 15 s, 20 s, and 25 s PAWs were mixed with MPs and named CK (0 s), PAW5s (5 s), PAW10s (10 s), PAW15s (15 s), PAW20s (20 s), and PAW25s (25 s), respectively.

### 2.4. Surface Hydrophobicity Measurement of Carbonyl Value

The surface hydrophobicity of MPs was determined using the bromophenol blue binding method (BPB), as described by Li et al. [10]. The concentration of MPs was diluted to 2 mg/mL. Moreover, 1 mL of the MPs was mixed with 40 μL of 1 mg/mL BPB solution and fully reacted at room temperature. After the reaction, the mixture was centrifuged at 6000 r/min, 4 °C for 15 min, and the supernatant was collected. The absorbance was tested at 595 nm using a UV spectrophotometer (TU1810, Beijing General Instrument Co., Ltd., Beijing, China).

(1) $$BPB\;bound\;(\mu g)=\frac{\left(40\;\mu L\times A\_control-A\_sample\right)}{A\_control}$$

### 2.5. Ultraviolet Spectroscopy

Deionized water was used as a blank, the concentration of MPs was diluted to 1 mg/mL. and the absorbance of sample was detected by UV spectrophotometer. The measurement range was from 200 to 360 nm.

### 2.6. Fluorescence Spectroscopy

The tertiary structure of MP was determined using fluorescence chromatography (F-7000, HITACHI, Tokyo, Japan). The concentration of MPs was diluted to 0.1 mg/mL. The settings of fluorescence spectroscopy parameters were as follows: excitation wavelength of 280 nm, emission wavelength range of 300–460 nm, slit width of 2.5 nm for all, and scanning rate of 12,000 nm/min.

### 2.7. Sodium Dodecyl Sulfate-Polyacrylamide Gel Electrophoresis (SDS-PAGE)

MP compositional changes were measured using SDS-PAGE under non-reducing and reducing conditions, respectively, as described by Liu et al. [11]. Under the reducing conditions, protein samples were mixed in equal volumes with loading buffer (0.5 mol/L Tris-HCl, 10% (*w*/*v*) SDS, 20% (*v*/*v*) glycine, 3.33% (*w*/*v*) dithiothreitol (DTT), 2% (*w*/*v*) bromophenol blue), boiled in water bath at 100 °C for 5 min, and centrifuged at 13,000 r/min for 1 min. After that, 5 μL of supernatant was loaded into the well. The concentrations of separating and concentrating gels were 10% and 5%. Under non-reducing conditions, there was no DTT in the loading buffer. Electrophoresis was performed at 70 V for separating gels and 110 V for concentrating gels. Gels were stained with Thomas Brilliant Blue R-250 for 30 min and then decolorized with 5% ethanol and 7.5% acetic acid. The gels were scanned, photographed, and analyzed using a gel imager (GS-900, BIO-RAD Laboratories, Inc., Hercules, CA, USA). Bands were analyzed using Image J software (1.8.0_172).

### 2.8. Total Sulfhydryl Content

The total sulfhydryl groups of MPs were measured using a total sulfhydryl measurement kit (BC5805, Beijing Solepol Technology Co., Ltd., Beijing, China). The concentration of MP solution was diluted to 5 mg/mL. According to the kit instructions, Tris-Gly buffer was added first and mixed thoroughly. Then, a certain amount of DTNB was added and mixed thoroughly, and the reaction was carried out while avoiding light at 4 °C. After the reaction, centrifugation (3000 r/min, 10 min) was performed. The supernatant was taken and added to a 96-well plate, and the absorbance was measured at 412 nm using an enzyme meter (20M/*, Tecan Schweiz AG., Männedorf, Switzerland).

### 2.9. Carbonyl Content

The carbonyl content of MPs was measured using the Protein Carbonyl Content Kit (BC1275, Beijing Soleberg Technology Co., Ltd. Beijing, China). The concentration of MPs was diluted to 5 mg/mL and then mixed with 2,4-dinitrophenylhydrazine (DNPH). The reaction was performed at room temperature, and light was avoided for 1 h. Then, trichloroacetic acid was added, centrifuged at 12,000 r/min, 4 °C for 15 min, and the supernatant was discarded. After that, precipitation was washed and centrifuged 3 times (12,000 r/min, 15 min, 4 °C) with a mixture of ethyl acetate/ethanol (1:1, *v*/*v*) dissolved in 6 mol/L guanidine hydrochloride solution and water-bathed at 37 °C. When the precipitation was completely dissolved, centrifugation was performed at 12,000 r/min for 10 min, and the supernatant was discarded. The absorbance was measured at 370 nm using an enzyme marker.

### 2.10. Secondary Structure of MPs

The secondary structure of MPs was detected using circular dichroism (Chirascan qCD, Applied Photophysics Ltd., Surrey, UK) as described by Jia et al. [12] with slight modifications. MPs were diluted to 0.1 mg/mL. Then, 1 mL of diluted MPs was taken for the test. Spectra was acquired by scanning in the wavelength range of 200–260 nm. The contents of the secondary structure were measured using CDNN software (cssetup_415). The instrument scan rate was 100 nm/min.

### 2.11. Preparation of MP Gels

MP gels were prepared as described by Shi et al. [13] with slight modifications. Six equal MP solutions were adjusted to 50 mg/mL with the prepared 0, 5, 10, 15, 20, and 25 s PAW, respectively. Then, 10 g of MPs were filled into a 10 mL beaker, sealed, placed in a water bath, and heated to 80 °C for 30 min. After heating, samples were immediately cooled in an ice bath for 10 min and placed in a 4 °C refrigerator for 12 h to obtain the MP gels.

### 2.12. Color and Whiteness

The color and whiteness of MP gels were detected, referring to the method of Wang et al. [14]. A fully automated colorimeter and a 10° field of view in 0/d mode were utilized to measure the color of gels. Before measurement, a standard color difference plate (standard values: L* = 94.81, a* = 100.00, b* = 107.32) was used to calibrate the colorimeter. After calibration, 6 random points were measured for each sample, and the L*, a*, b* and whiteness (W) of the MP gels were recorded. The average of the six measurements was taken. Gel whiteness was calculated following the formula below:

(2) $$Gel\;whiteness=100-\sqrt{{\left(100-L^{*}\right)}^{2}+{a^{*}}^{2}+{b^{*}}^{2}}$$

### 2.13. Gel Strength

Gel strength was determined using a TA.XT. plus texture analyzer equipped with a cylindrical probe (P/0.5R) (TA.XT. plus, Stable Micro Systems Co., Ltd., Godalming, UK), referring to the method of Li et al. [10]. The speed was set to 2.0 mm/s before and after the test and 1.0 mm/s during the test. The trigger force was 5 g, and the compression deformation was 50%. Each set of samples was measured 6 times.

### 2.14. Textural Properties

Textural properties, including hardness, resilience, recoverability, adhesiveness, cohesiveness, and chewability of MP gels, were tested as described by Li et al. [15] with slight modifications. MP gels were split into equally sized cylinders (2.0 cm thickness, 2.5 cm diameter). A texture analyzer equipped with a P/50 probe (TA.XT. plus, Stable Micro Systems Co., Ltd., Godalming, UK) was used, and the speed before, during, and after the test was 2, 1, and 2 mm/s, respectively. A double compression cycle test was performed with a compression percentage of 50% of the height of the sample.

### 2.15. Moisture Distribution

The internal moisture distribution of MP gels was detected using low-field nuclear magnetic resonance (LF-NMR), as described by Cheng et al. [16]. Samples (~2 g) were put in 2 mL cylindrical tubes and then scanned using an LF-NMR analyzer (NM120, Niumag Co., Ltd., Suzhou, China). The transverse relaxation times of the samples were recorded using the Carr-Purcell-Meiboom-Gill (CMPG) sequence T2. Parameter settings were as follows: sampling frequency was 200 KHz, the echo count was 15,000, the time between 90° and 180° pulses was 13 us, the scanning range was 0–10,000 ms, and there were 4 repetitions. Finally, samples were tested with the multi-spin echo pulse sequence imaging.

### 2.16. Secondary Structure of MPs in Gels

Fourier transform infrared spectroscopy (FTIR) (Vertex 70, Bruker Co., Ltd., Berlin, Germany) was used to analyze the secondary structure of MPs in gels as described by Sun et al. [17]. Prepared gel samples were frozen with liquid nitrogen and then freeze-dried using a freeze dryer. Then, KBr was mixed with dried gel samples in a ratio of 100:1 (*w*/*w*), ground into powder with a special mortar, and pressed into thin tablets using a tablet press for infrared determination. Measurement parameters were set as follows: the scanning time was 32 s, resolution was 4 cm^−^^1^, and the scanning range was 4000–400 cm^−^^1^. The results of the scans were deconvoluted with Gaussian fitting using Peak fit 4.12 software to analyze the relative content of the secondary structure of MP in gels.

### 2.17. Molecular Forces

Molecular forces were detected according to the method of Xiong et al. [18] with minor modifications. Two grams of the 5 prepared MP gel samples, weight 2 g, were taken and added to 10 mL of the following reagents: S1: 0.05 mol/L NaCl; S2: 0.6 mol/L NaCl; S3: 0.6 mol/L NaCl + 1.5 mol/L Urea; S4: 0.6 mol/L NaCl + 8 mol/L Urea; and S5: 0.6 mol/L NaCl + 8 mol/L Urea + 0.5 mol/L β-mercaptoethanol, respectively. After that, the sample was homogenized at 10,000 r/min and allowed to stand at 4 °C for 1 h. After the reaction, centrifugation was performed at 10,000× *g* for 15 min. The supernatant was taken, and the protein concentration was measured using the bisulfite method. Molecular forces were calculated following the formula below:

Ionic bond content = S2 − S1.

Hydrogen bonding content = S3 − S2.

Hydrophobic interaction content = S4 − S3.

Disulfide bond content = S5 − S4.

### 2.18. Microstructure

High-resolution cold-field scanning electron microscopy (FE-SEM) (Regulus 8100, HITACHI, Tokyo, Japan) was used to observe the microstructure of MP gels according to the method of Zhou et al. [19]. Prepared MP gels were sliced into small pieces and placed in special sample trays for FE-SEM. Liquid nitrogen was used for rapid freezing of the samples. The samples were transported into the preparation chamber using the instrument’s own transportation bar, and the surface of the samples was sliced using a special knife at very low temperatures. Sublimation was carried out at −75 °C for 15 min, followed by gold sputtering at 10 mA for 60 s. Then, the sample was transported to the observation chamber. The microstructure of the MP gels was photographed under an accelerating voltage of 3 kV at magnifications of 1000× and 3000×, respectively.

### 2.19. Statistical Analysis

All experiments were carried out at least 3 times in parallel. Data were analyzed by one-way analysis of variance (ANOVA) with SPSS 25.0 software (SPSS 25.0 for Windows, SPSS Inc., Chicago, IL, USA). The results of the experiment were presented as mean ± standard deviation. Figures were drawn using Origin 2023 (Origin Lab Co., Northampton, MA, USA). Significance was analyzed using Duncan’s multiple comparison test (*p* < 0.05).

## 3. Results and Discussion

### 3.1. Surface Hydrophobicity

**Figure 1:**
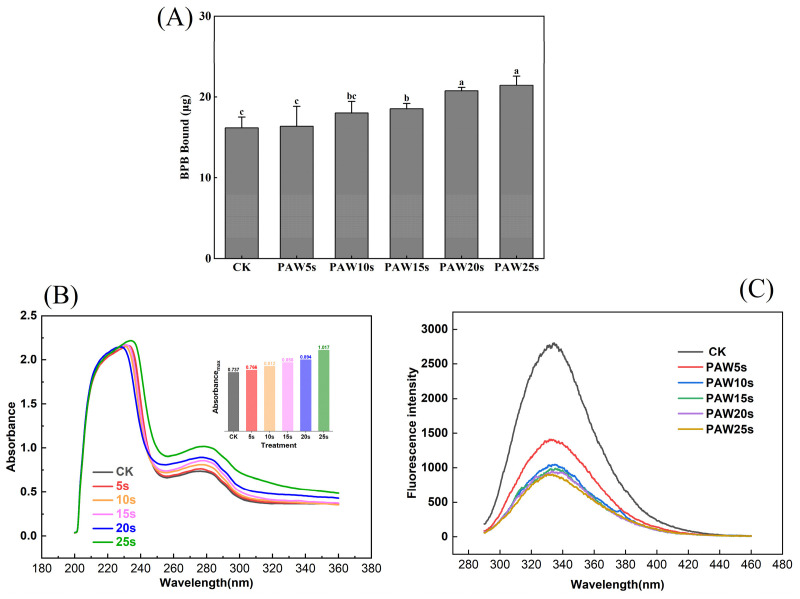
Effect of different PAW treatments on the surface hydrophobicity (**A**), ultraviolet absorption spectrum (**B**) and fluorescence spectrum (**C**) of MPs extracted from thawed pork. Different lower cases indicate significant differences (*p* < 0.05). CK indicates control. PAW5s, PAW10s, PAW15s, PAW20s, and PAW25s indicate MP treated with the prepared 0 s, 5 s, 10 s, 15 s, 20 s, and 25 s PAW, respectively.

BPB bound reflects surface hydrophobicity of protein; the higher BPB bound, the stronger the surface hydrophobicity of protein [10] (Li et al., 2019). As presented in Figure 1A, the surface hydrophobicity of MPs increased after PAW treatment. PAW obtained with the longer plasma treatment presented a greater ability to enhance the surface hydrophobicity of MPs. Compared with the control group, surface hydrophobicity was significantly higher in the PAW15s, PAW20s, and PAW25s groups (*p* < 0.05). A maximum value of 21.5 μg was observed in the PAW25s group, but the difference was not significant compared with the PAW20s group (*p* > 0.05). The large amounts of H_2_O_2_ and O_2_ contained in PAW accelerate the oxidation of MPs to some extent, resulting in higher exposure of hydrophobic groups [20]. Previous research has shown that a gradual increase of ROS radicals and hydroxyl radicals in PAW would attack the surface of proteins, expose hydrophobic amino side chains, and further improve the surface hydrophobicity of proteins [21,22]. Furthermore, Li et al. [6] found that as PAW treatment time increased, the surface hydrophobicity of MPs increased accordingly, which is consistent with our results.

### 3.2. Ultraviolet Spectroscopy

Tryptophan (Trp) and tyrosine (Tyr) residues in proteins could absorb UV light, and the absorption intensity is usually used to reflect the changes in protein conformation. As shown in Figure 1B, after PAW treatment, the UV absorption intensity of MP was significantly enhanced from 0.7 to 1.0 (*p* < 0.05), which showed a positive correlation with the PAW treatment time. Previous research has found that the UV absorption intensity of PAW-treated duck MP significantly increased as the generation time of PAW increased, leading to higher exposure of hydrophobic groups and the corresponding enhancement of cross-linking between proteins [23], which is consistent with our results. In addition, in the PAW25s group, the maximum absorption wavelength shifted from 274 to 278 nm, indicating that with the increased PAW generation time, more hydrophobic groups were exposed and the polarity of proteins switched at this time. In this case, the interactions between MPs enhanced and further promoted the aggregation of MPs [24]. Qian et al. [7] used PAW to treat MPs of chicken and found that MPs treated with PAW showed higher UV absorbance than untreated MPs, and the maximum absorption wavelength of MPs shifted from 287 nm to 297 nm after PAW treatment, reflecting the fact that more hydrophobic groups of MPs were exposed.

### 3.3. Intrinsic Fluorescence Spectroscopy

Fluorescence energy in the microenvironment easily induces changes in the intrinsic fluorescence of tryptophan and tyrosine residues in proteins. Changes in fluorescence intensity respond to the changes in protein tertiary structure [25]. As presented in Figure 1C, PAW-treated MPs underwent a significant decrease in fluorescence intensity (*p* < 0.05) compared with the control group. In addition, the fluorescence intensity continuously decreased with the prolongation of the PAW generation time. Studies have verified that lower fluorescence intensity accompanied by red-shifted fluorescence spectra indicates a higher oxidation degree of protein [24]. In this study, MPs treated with PAW showed significantly lower fluorescence intensity, reflecting that PAW deepened the oxidation of MPs. These results also correspond to the results of surface hydrophobicity of MPs. The decreased in fluorescence intensity caused by the oxidation of amino acid residues in MPs, resulting in the breakage of polypeptide chains and the formation of cross-linked protein aggregates. With fewer tyrosine and tryptophan residues exposed, the polarity to the fluorescence energy of the microenvironment attenuated [20].

### 3.4. SDS-PAGE

**Figure 2:**
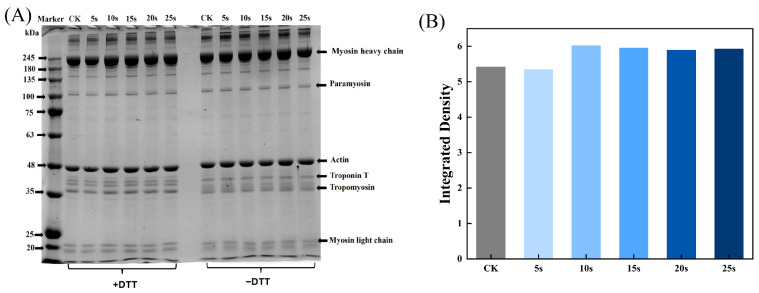
Effect of PAW treatments on SDS-PAGE (**A**) and relative band intensity (**B**) of MPs extracted from thawed pork. CK, control.

The aggregation and degradation of MPs after PAW treatments were determined by SDS-PAGE. As presented in Figure 2A, there were two clearly visible bands at 45 kDa and 200 kDa, corresponding to actin and myosin heavy chains, respectively. In addition, bands of myosin light chains (16–25 kDa), troponin (35 kDa), and troponin T (38 kDa) were also detected. The intensity of myosin-heavy chains in reductive SDS-PAGE was analyzed using Image J software and is presented in Figure 2B. The results showed that the composition of MPs remained unchanged after PAW treatments, but in the non-reducing condition, the band intensity of the PAW25s group weakened. This was likely due to the formation of the aggregates in the PAW25s group, possibly as a result of the formation of the interactions between the carbon–carbon covalent bonds of MPs induced by protein oxidation [26]. In a non-reduced environment, with the increasing time of PAW generation, a clearly upward trend of band intensities in the range of 180 kDa to 245 kDa was found. DTT, as a kind of redox reagent, could completely or partially disrupt disulfide bonds in proteins, thereby affecting protein structure and functions [27]. The bands’ intensity with a molecular weight of more than 245 kDa became lighter after the addition of DTT, demonstrating that more protein aggregates cross-linked by disulfide bonds were degraded. Xiong et al. [28] found that the addition of a reductive agent (β-mercaptoethanol) to MP solutions containing H_2_O_2_ resulted in the higher intensity of MHC bands, and the intensity increased as the amount of H_2_O_2_ increased due to the ability of H_2_O_2_ to promote the disulfide formation between proteins. Therefore, in this study, PAW also promoted cross-linking of MPs through disulfide bonds, and the higher degree of MPs aggregation after PAW treatment may be attributed to the lower degree of protein degradation.

### 3.5. Total Sulfhydryl Content

**Figure 3:**
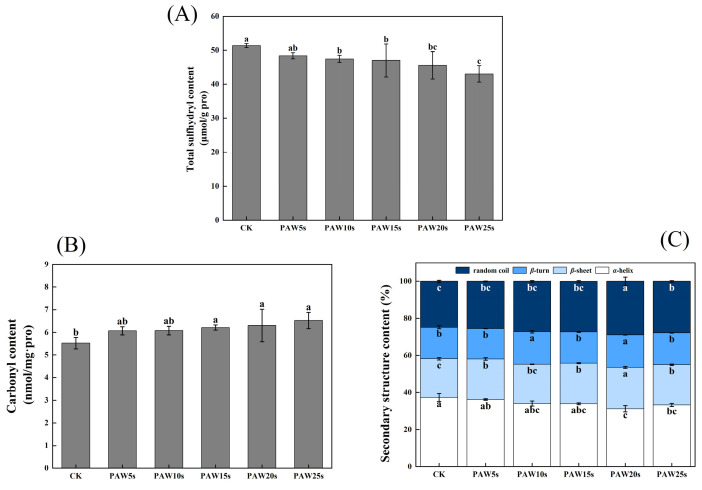
Effect of PAW treatments on total sulfhydryl content (**A**), carbonyl content (**B**) and secondary structure (**C**) of MPs extracted from thawed pork. Different lower cases indicate significant differences (*p* < 0.05). CK represents the control. PAW5s, PAW10s, PAW15s, PAW20s, and PAW25s represent MP treated with the prepared 0 s, 5 s, 10 s, 15 s, 20 s, and 25 s PAW, respectively.

Sulfhydryl groups (SH) contained in MP are highly sensitive to oxidation and then turn into disulfide bonds (S-S) [29]. The reduction in SH content represents the fact that the SH group of cysteine is oxidized and the S-S is increased. As shown in Figure 3A, the total sulfhydryl content of MPs decreased significantly after PAW treatment (*p* < 0.05) and reached a minimum value of 43.0 μmol/g-pro in the PAW25s group. However, except for PAW25s, the total sulfhydryl content showed no significant differences between the other PAW groups (*p* > 0.05). Cao et al. [25] investigated the oxidation of MPs and found that the SH groups in actin were sensitive to hydroxyl radicals and were easily converted to intermolecular S-S bonds after oxidation. In addition, Fe^3^⁺ catalyzes the generation of hydroxyl radicals from H_2_O_2_, and to some extent, Fe^3^⁺-induced oxidation has a synergistic effect with PAW.

### 3.6. Carbonyl Content

Carbonyl groups are the main oxidation products of tryptophan, proline, arginine, and lysine residues in proteins. Proteins with -NH and -NH_2_ groups in side chains are easily deamidated to form carbonyl derivatives [30]. As shown in Figure 3B. The carbonyl content was 5.77 nmol/mg pro in the control group. Compared with the control group, carbonyl content in the PAW15s, 20s, and 25s groups significantly increased (*p* < 0.05) and reached a maximum value of 6.52 nmol/mg pro in the PAW25s group. PAW5s and PAW10s group had higher carbonyl content than the control group but showed no significant differences (*p* > 0.05). The results indicated that the oxidation degree of MPs was not significantly influenced by PAW treatment with short generation time (5 s, 10 s) but was further improved with the prolonged generation time of PAW. The impact of high-energy particles in the PAW solution also disrupts the structure of MPs, causing more residues of MPs to be exposed and further promoting the carbonylation of protein residues [31]. PAW treatment, to some extent, improved the oxidation of MPs, which corresponded to the previous results of the total sulfhydryl content of MPs.

### 3.7. Secondary Structure

Figure 3C shows the changes in the secondary structure of MP. After PAW treatment, the α-helix, β-turn, β-sheet and random coil content of MP changed perceptibly. Among them, the α-helix content generally presented a decreasing trend and reached the minimum value of 31.25% in the PAW20s group, which was significantly lower than that in other groups (*p* < 0.05). The β-sheet and random coil content gradually increased, accompanied by the generation time of PAW increased to 20 s, and then decreased at 25 s generation time. However, compared with the control group, random coil and β-sheet content in the PAW25s group was significantly higher (*p* < 0.05). The PAW5s, PAW15s, and PAW25s groups presented no significant differences in β-turn content compared with the control group (*p* > 0.05), while β-turn content in PAW10s and PAW20s groups was significantly higher than that in the control group (*p* < 0.05). Usually, proteins maintain their biologically active structures in vitro through weakening interactions such as hydrophobic interactions, hydrogen bonding, and ionic bonding. When the surroundings change, these interactions may be altered, leading to protein denaturation and inactivation. Hydrogen bonds formed between the amino hydrogen (-NH_2_) and carbonyl oxygen (-CO) in the peptide chain help stabilize the α-helix structure [32]. PAW contains a high content of active substances such as NO_3_^−^, NO^−^, and RONS. These substances could weaken the interaction between proteins and water, break hydrogen bonds, and promote the α-helix structure to deconvolute and transform into other structures [33]. Therefore, the further prolonged of the PAW treatment time decreased the α-helix content in this study. A certain degree of PAW treatment decreased the α-helix content of protein, resulting in more exposure of hydrophobic groups, more aggregation of proteins, and more tightly linked between proteins [34], which corresponded to the previous results of surface hydrophobicity of MPs.

### 3.8. Color and Whiteness of MP Gels

Color is a very important sensory indicator for meat products and gels. As presented in Table 1, with the increased PAW generation time, L*, a*, b*, and whiteness showed a trend of first increasing and then decreasing. All of these indexes reached the maximum value in the PAW20s group. The whiteness of the gels increased from the initial 76.35 to 85.06. Usually, the whiteness value of gel is closely related to gel quality [35]. PAW treated groups presented significantly higher whiteness than the control group (*p* < 0.05), demonstrating that gels prepared by PAW treated MPs might contain higher content of moisture, which enhances the light reflection of gels. PAW treatment may help increase the water content in the network structure of MP gels.

### 3.9. Gel Strength

**Figure 4:**
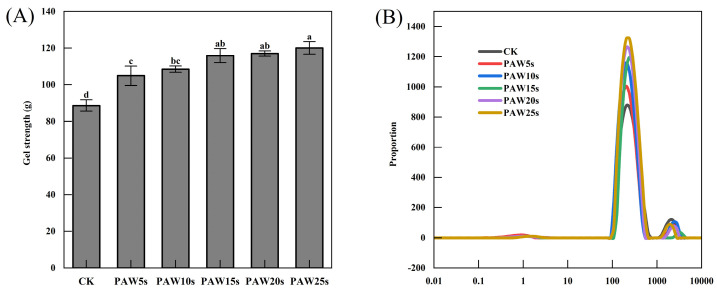
Effect of PAW treatments on gel strength (**A**) and moisture distribution (**B**) of MP gels. Different lower cases indicate significant differences (*p* < 0.05). CK indicates the control. PAW5s, PAW10s, PAW15s, PAW20s, and PAW25s indicate MP treated with the prepared 0 s, 5 s, 10 s, 15 s, 20 s, and 25 s PAWs, respectively.

The strength of MP gels is presented in Figure 4A. A gradual increase in gel strength was observed with the increase in PAW treatment time. The control group had the smallest gel strength of 88.6 g (*p* < 0.05). Compared with the control group, MP gels in the PAW treatment groups significantly increased (*p* < 0.05) and reached a maximum value of 120.1 g in the PAW25s group. Previous research has proven that the adequate oxidative stress induced by reactive oxygen species in PAW increases the intramolecular and intermolecular interactions of MPs, contributing to the improvement of MP gel strength [36]. It is consistent with our results. A significant improvement of MP gel strength was observed after MPs were treated with PAW, indicating a positively role of PAW in the improvement of MP gel quality.

### 3.10. Moisture Distribution

As a non-destructive method, LF-NMR has been widely used to evaluate the distribution and migration of water in protein gels [37]. Three types of water are reflected by three characteristic peaks, respectively: T_2b_ (0.1–10 ms, bound water firmly bonds to the protein gel), T_21_ (10–1000 ms, immobilized water limited by the structure of the protein gel), and T_22_ (1000–10,000 ms, free water located outside the protein gel). The results in this study are in accordance with the relaxation time of meat gels as described by Wang et al. [38] and Zhang et al. [39]. As shown in Figure 4B, there were no significant differences in T_2b_ relaxation time among all groups (*p* > 0.05), but a significant change in the relaxation time of T_21_ and a significant increase in the peak amplitude of T_21_ in PAW groups was observed compared with the control group (*p* < 0.05). Li et al. [40] found that PAW treatments promoted a decrease in T_22_ and an increase in T_21_ of MP gels, reflecting a shift from free water to immobilized water in gels. Proteins cross-linked gradually after PAW treatment and finally acted on the formation of a robust gel network structure that can trap more water. Therefore, the results of our study demonstrate that PAW treatment improved the water retention of the gels. PAW might enhance the intermolecular forces between MPs and promote the formation of gels with homogeneous and tight network structures. However, the differences in the proportion of peak area between different PAW groups were insignificant (*p* > 0.05). This may be due to the short generation time of the PAW. The relaxation time of T_22_ in the PAW groups was significantly lower than that in the control group (*p* < 0.05), also illustrating the fact that gels in the PAW groups had less content of free water and higher water-holding capacity. The peak area ratios of T_22_, T_21_, and T_2b_ were displayed as P_22_, P_21_, and P_2b_, respectively, and are presented in Table 2. As shown in Table 2, there were no significant differences (*p* > 0.05) between the P_2b_ of the groups, demonstrating that the bound water in gels was unaffected by PAW treatment. However, compared with the control group, P_21_ and P_22_ in the PAW groups significantly increased and significantly decreased (*p* < 0.05), respectively. In MP gels, the content of immobilized water increased significantly, and the content of free water decreased significantly after MPs were treated with PAW. Differences of P_21_ and P_22_ were insignificant between different PAW groups, which might due to the short duration of PAW treatment and was corresponding to the results of T_2b_, T_21_, and T_22_.

### 3.11. Textural Properties

**Figure 5:**
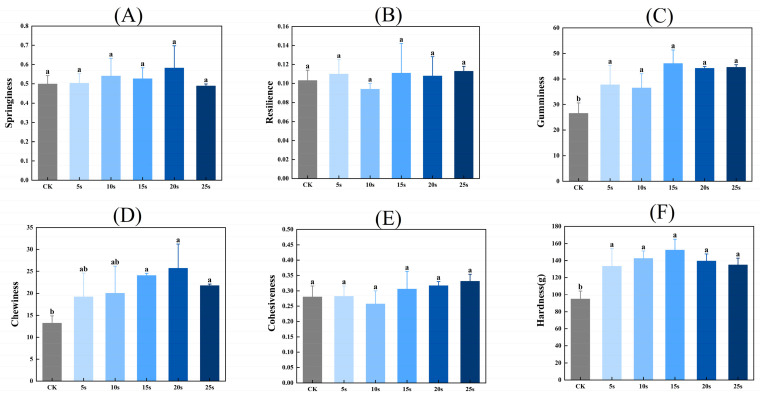
Effect of PAW treatments on the textural properties of MP gels. (**A**–**F**), springiness (**A**), resilience (**B**), gumminess (**C**), chewiness (**D**), cohesiveness (**E**), and hardness (**F**). Different lower cases indicate significant differences (*p* < 0.05). CK indicates the control. PAW5s, PAW10s, PAW15s, PAW20s, and PAW25s indicate MPs treated with the prepared 0 s, 5 s, 10 s, 15 s, 20 s, and 25 s PAWs, respectively.

Figure 5 presents the textural properties of MP gels. As shown in Figure 5, the hardness, chewiness, and adhesiveness of gels were significantly higher in PAW groups compared with the control group (*p* < 0.05). However, the resilience and cohesiveness of gels presented insignificant differences between all groups (*p* > 0.05). PAW improves the solubility of MPs before heating, which provides more opportunities for adequate interactions between proteins and further contributes to the formation of protein gels with higher textural properties [41]. Furthermore, Luo et al. [42] found that plasma could promote the emulsification properties of MPs, which was closely positively related to the gel properties of MPs.

### 3.12. FTIR

**Figure 6:**
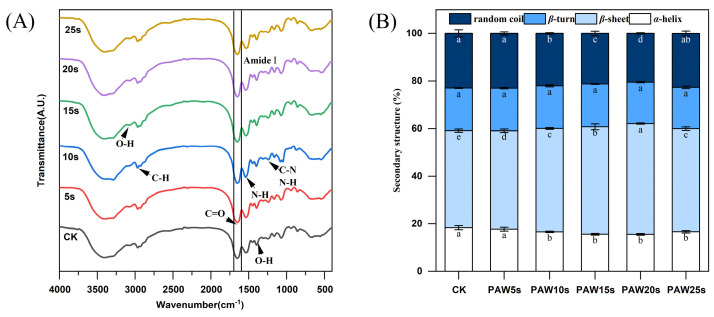
Effect of PAW treatments on secondary structure of MP in gels. (**A**,**B**), FTIR spectra (**A**), secondary structure content (**B**). Different lower cases indicate significant differences (*p* < 0.05). CK indicates control. PAW5s, PAW10s, PAW15s, PAW20s, and PAW25s indicate MPs treated with the prepared 0 s, 5 s, 10 s, 15 s, 20 s, and 25 s PAWs, respectively.

The secondary structure of MP in gels was determined using Fourier infrared spectroscopy (FTIR). Figure 6A shows the FTIR spectra in the range of 400–4000 cm^−^^1^. The amide I band (1600–1700 cm^−^^1^) is a typical absorption peak, which is mainly caused by C=O stretching vibration, N-H bending vibration and C-N stretching vibration, and is often used to characterize secondary structure changes of MP in gels. Absorption peaks correspond to the secondary structure of the protein as follows: β-sheet 1600 to 1640 cm^−^^1^, random coil 1640 to 1650 cm^−^^1^, α-helix 1650 to 1660 cm^−^^1^, and β-turn 1660 to 1700 cm^−^^1^ [43]. Figure 6B presents the α-helix, β-turn, β-sheet, and random coil content of the MPs in gels. Except for the PAW5s group, the α-helix content in the other PAW groups was significantly lower compared with the control group (*p* < 0.05) and reached a minimum in the PAW20s group. The β-sheet content in PAW groups, except for PAW25s, was significantly higher compared with the control group (*p* < 0.05). However, there were no significant differences in β-turning content between all groups (*p* > 0.05). It is generally recognized that during the thermal induction of proteins into the gels process, α-helix are converted to β-turn and β-sheet, and a higher β-sheet content represents a better secondary structure of proteins in gels [44]. Furthermore, ROS, hydroxyl radicals, and other reactive substances consisting in PAW contribute to the increased conversion of α-helix during the formation of thermal-induced MP gels [45]. Therefore, the results of this study confirm and indicate that the previous PAW treatment of MPs promoted the conversion from α-helix to β-sheet of MPs during the formation of thermal-induced gels.

### 3.13. Molecular Forces

**Figure 7:**
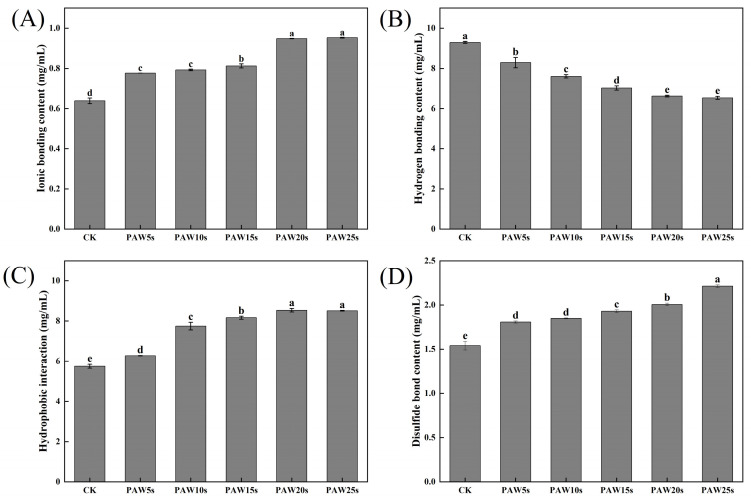
Effect of PAW treatments on molecular forces in MP gels. (**A**–**C**), ionic bonding content (**A**), hydrogen bonding content (**B**), hydrophobic interaction (**C**), and disulfide bond content (**D**). different lower cases indicate significant differences (*p* < 0.05). CK indicates the control. PAW5s, PAW10s, PAW15s, PAW20s, and PAW25s indicate MPs treated with the prepared 0 s, 5 s, 10 s, 15 s, 20 s, and 25 s PAWs, respectively.

The detection of intermolecular forces in MP gels provides insight into the interactions between molecules that occur in MP gels [46]. As presented in Figure 7, as the generation time increases, the ionic bonding content of PAW groups significantly increases (*p* < 0.05) compared with the control group, reaching the maximum value of 49.11% in the PAW25s group. Hydrogen bonding content decreased significantly in PAW groups compared with the control group (*p* < 0.05), reaching the minimum in the PAW25s group, with a reduction of 29.68%, indicating that PAW significantly enhanced the breakage of hydrogen bonds. The hydrophobic interactions showed a significant increase of 48.11% with the extension of PAW treatment time (*p* < 0.05). Moreover, the disulfide bonds of gels significantly increased (*p* < 0.05) by 43.72% after PAW treatment. The increased oxidation of MPs after PAW treatment promotes sulfhydryl groups in MPs to form more disulfide bonds during gel formation, leading to the obvious increase of the disulfide bond content in all PAW treatments. Hydrophobic interactions play an important role in the formation of gels, and the disulfide bonds are closely related to the three-dimensional structure of proteins in gels [47]. Thus, the results of this study demonstrate that the application of PAW was beneficial to the formation of gels with stable structures, contributing to the enhancement of gel properties of MPs.

### 3.14. Microstructure

**Figure 8:**
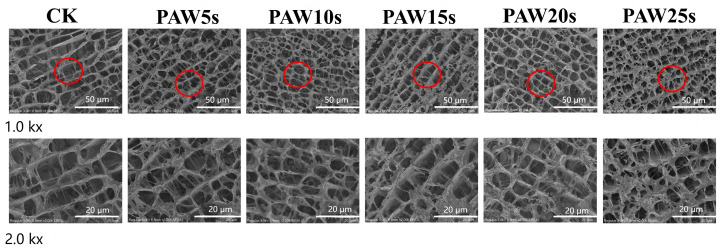
Effect of PAW treatments on the microstructure of MP gels. CK indicates the control. PAW5s, PAW10s, PAW15s, PAW20s, and PAW25s indicate MPs treated with the prepared 0 s, 5 s, 10 s, 15 s, 20 s, and 25 s PAWs, respectively.

The observation of the microstructure of MP gels was performed using high-resolution cold-field scanning electron microscopy (FE-SEM) and is presented in Figure 8. As shown in Figure 8, the MP gels in all groups showed macroscopically homogeneous structures similar to those of the typical carnosine gels. Compared with the control group, the microstructures of PAW-treated MP gels were denser and more homogeneous. MPs treated with PAW undergone more oxidative denaturation, resulting in more exposure of sulfhydryl groups and hydrophobic groups of MPs. Furthermore, the heat-induced gelation results in the cross-linking of exposed hydrophobic groups and the formation of disulfide bonds through sulfhydryl groups [48]. Therefore, the stronger protein interactions in PAW-treated gels contribute to the formation of gels with homogeneous microstructures and stronger texture properties. In other words, PAW treatment dispersed the protein aggregates, enhanced protein–water interactions, and, finally, formatted denser gels. Particularly, MP gels of PAW20s and PAW25s groups presented the best microstructures.

## 4. Conclusions

PAW treatment promoted the partial oxidation of MPs and generated more disulfide bonds in MPs. The changes in amino acid polarity and the exposure of hydrophobic groups in PAW-treated MPs also promoted intra-protein and protein–protein interactions. The enhanced properties of PAW-treated MPs of thawed pork promoted the formation of MP gels with more stable and dense structures. In particular, PAW with 20 s generation time significantly improved the strength, textural properties, and water-holding capacity of the MP gels. To sum up, PAW treatment altered the intermolecular forces and the structures of MPs extracted from thawed pork and helped improve the gel properties of MPs extracted from thawed pork significantly. This study developed a new method to improve the processing functions of MPs of thawed meat and may contribute to reducing weight losses of thawed meat products.

## Figures and Tables

**Table 1:** Effect of PAW on the color and whiteness of MP gels.

	L*	a*	b*	W
CK	76.75 ± 1.26 ^d^	−2.45 ± 0.17 ^c^	−3.52 ± 0.64 ^c^	76.35 ± 1.32 ^d^
PAW5s	80.32 ± 0.38 ^c^	−2.38 ± 0.02 ^c^	−2.59 ± 0.05 ^abc^	80.01 ± 0.36 ^c^
PAW10s	81.47 ± 0.53 ^c^	−1.87 ± 0.11 ^b^	−3.32 ± 0.48 ^bc^	81.08 ± 0.43 ^c^
PAW15s	83.03 ± 0.36 ^b^	−1.83 ± 0.04 ^b^	−2.54 ± 0.44 ^abc^	82.74 ± 0.32 ^b^
PAW20s	85.28 ± 0.63 ^a^	−1.59 ± 0.02 ^a^	−1.89 ± 0.41 ^a^	85.06 ± 0.59 ^a^
PAW25s	83.11 ± 0.79 ^b^	−2.04 ± 0.14 ^b^	−2.38 ± 0.81 ^ab^	82.82 ± 0.85 ^b^

Note: Different letters indicate the significance within each column (*p* < 0.05). CK indicates the control. PAW5s, PAW10s, PAW15s, PAW20s, and PAW25s indicated MP treated with the prepared 0 s, 5 s, 10 s, 15 s, 20 s, and 25 s PAWs, respectively.

**Table 2:** Effect of PAW on the ratio of different types of water in MP gels.

	P_2b_	P_21_	P_22_
CK	0.825 ± 0.195 ^a^	92.573 ± 0.229 ^b^	6.662 ± 0.128 ^a^
PAW5s	0.867 ± 0.783 ^a^	94.798 ± 0.310 ^a^	4.335 ± 0.493 ^b^
PAW10s	0.700 ± 0.056 ^a^	96.287 ± 0.173 ^a^	3.014 ± 0.115 ^b^
PAW15s	0.797 ± 0.128 ^a^	96.815 ± 0.130 ^a^	2.389 ± 0.009 ^b^
PAW20s	0.698 ± 0.142 ^a^	95.965 ± 0.231 ^a^	3.336 ± 0.368 ^b^
PAW`25s	0.749 ± 0.147 ^a^	95.362 ± 2.428 ^a^	3.889 ± 2.459 ^b^

Note: Different letters indicate the significance within each column (*p* < 0.05). CK indicates the control. PAW5s, PAW10s, PAW15s, PAW20s, and PAW25s indicate MPs treated with the prepared 0 s, 5 s, 10 s, 15 s, 20 s, and 25 s PAWs, respectively.

## Data Availability

The original contributions presented in the study are included in the article, further inquiries can be directed to the corresponding author.
